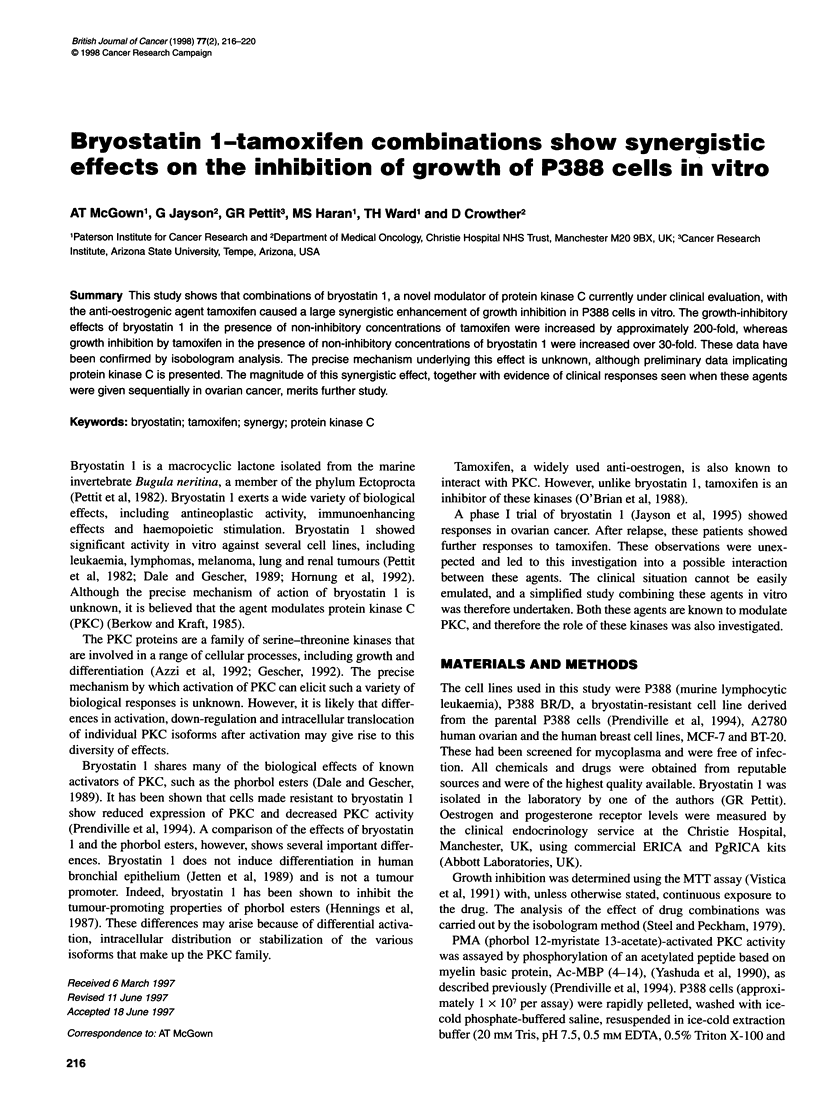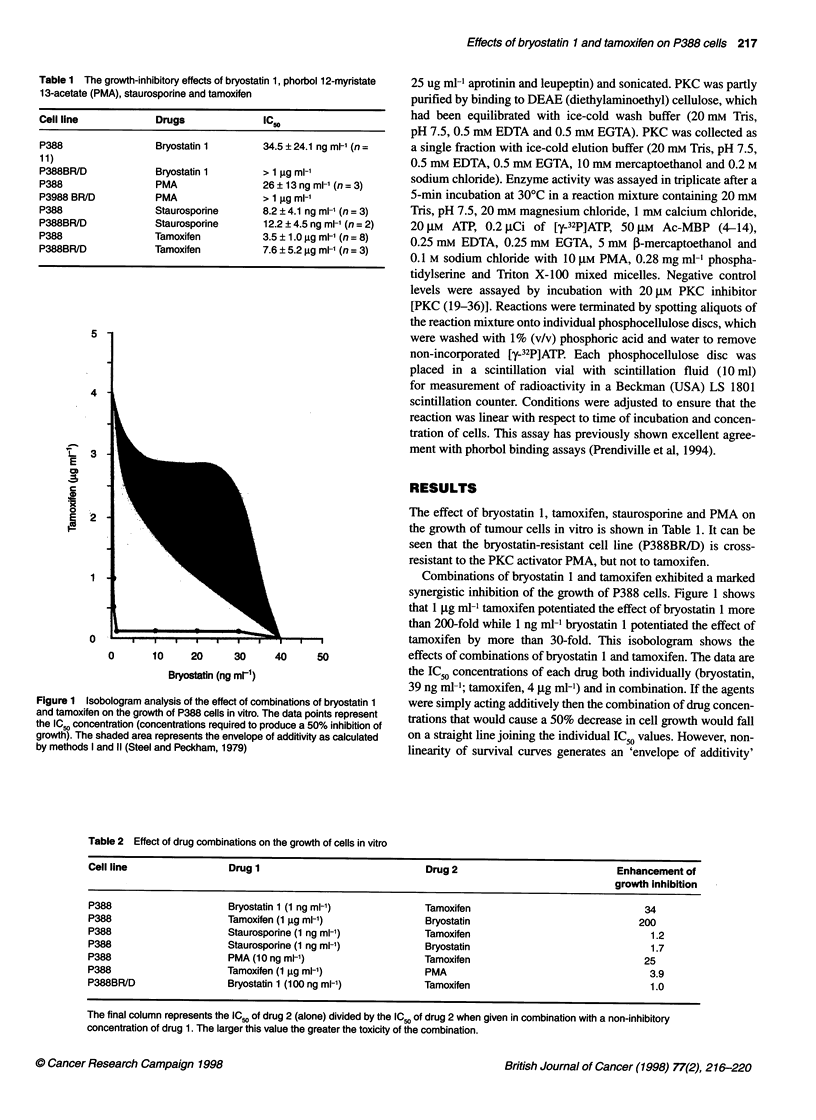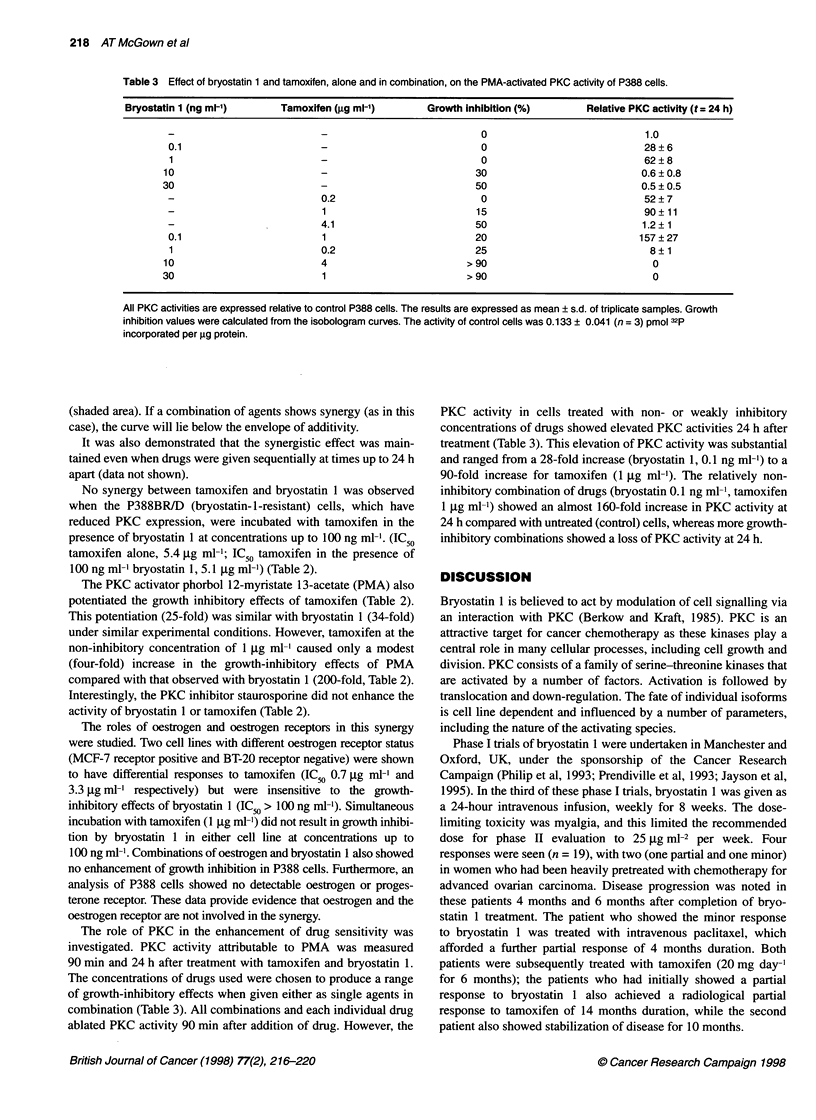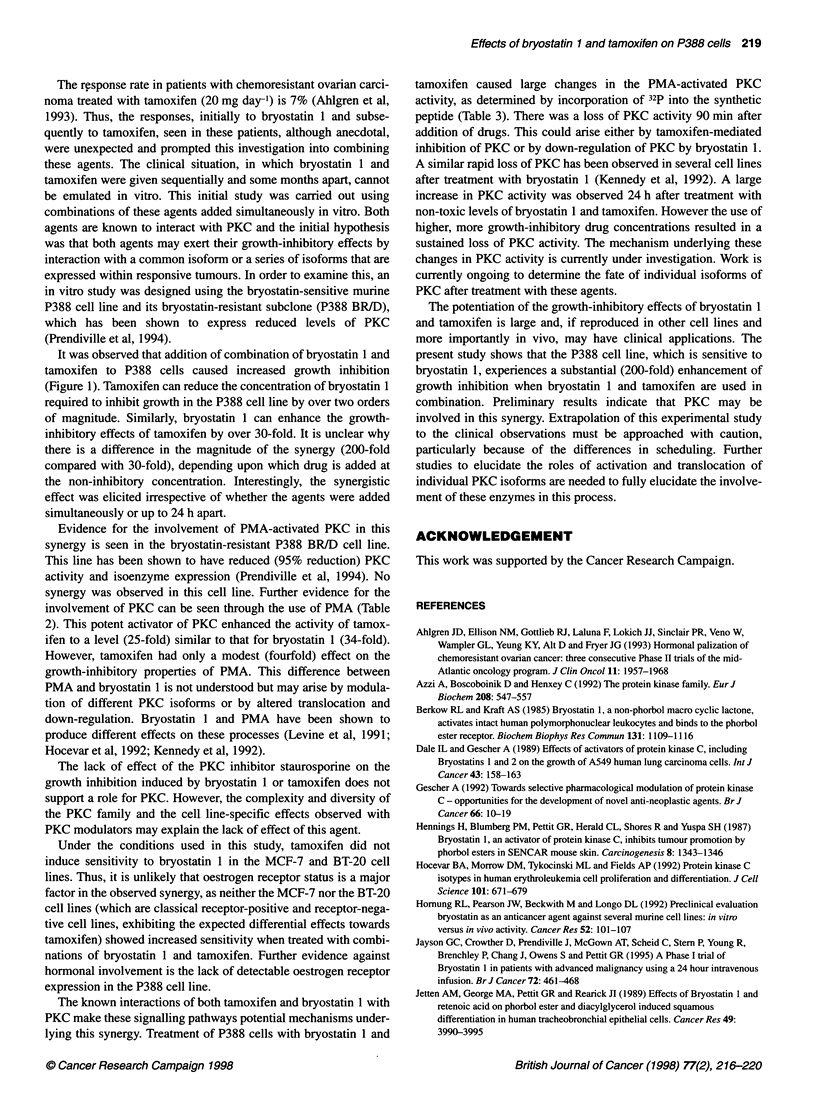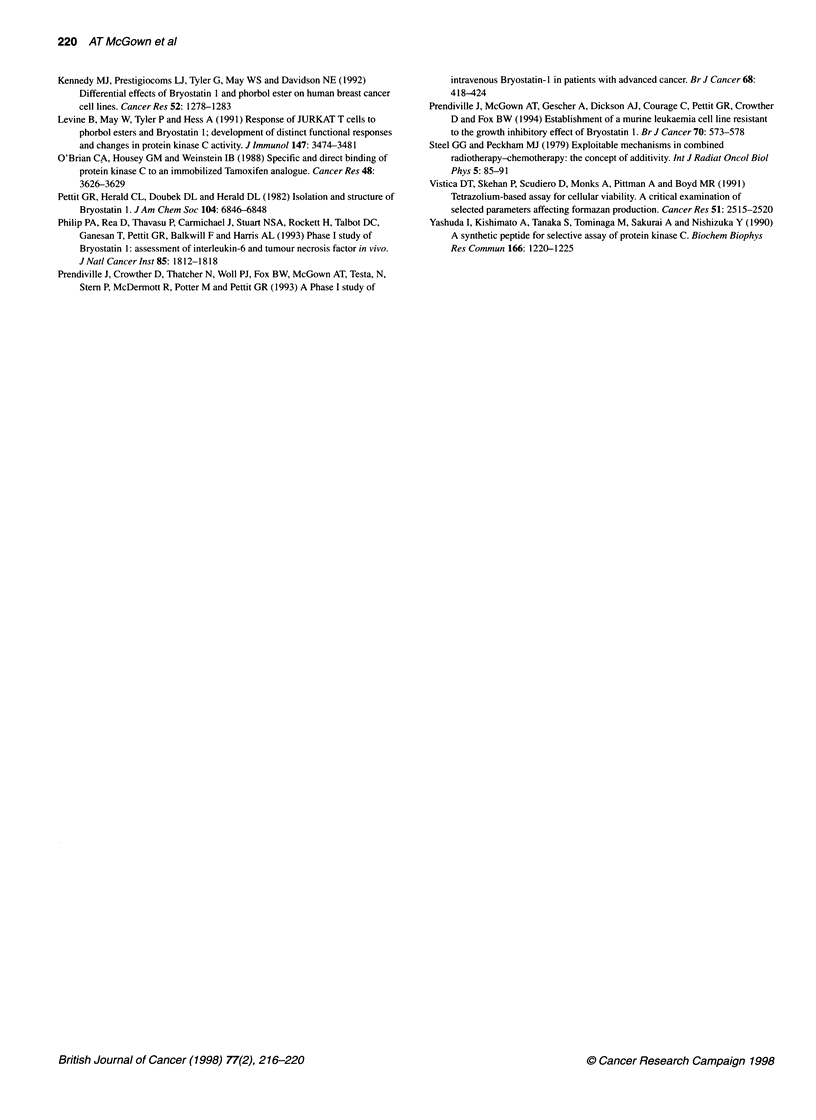# Bryostatin 1-tamoxifen combinations show synergistic effects on the inhibition of growth of P388 cells in vitro.

**DOI:** 10.1038/bjc.1998.36

**Published:** 1998

**Authors:** A. T. McGown, G. Jayson, G. R. Pettit, M. S. Haran, T. H. Ward, D. Crowther

**Affiliations:** Paterson Institute for Cancer Research, Christie Hospital NHS Trust, Manchester, UK.

## Abstract

This study shows that combinations of bryostatin 1, a novel modulator of protein kinase C currently under clinical evaluation, with the anti-oestrogenic agent tamoxifen caused a large synergistic enhancement of growth inhibition in P388 cells in vitro. The growth-inhibitory effects of bryostatin 1 in the presence of non-inhibitory concentrations of tamoxifen were increased by approximately 200-fold, whereas growth inhibition by tamoxifen in the presence of non-inhibitory concentrations of bryostatin 1 were increased over 30-fold. These data have been confirmed by isobologram analysis. The precise mechanism underlying this effect is unknown, although preliminary data implicating protein kinase C is presented. The magnitude of this synergistic effect, together with evidence of clinical responses seen when these agents were given sequentially in ovarian cancer, merits further study.


British Journal of Cancer (1998) 77(2), 216–220
© 1998 Cancer Research Campaign

### Bryostatin 1-tamoxifen combinations show synergistic effects on the inhibition of growth of P388 cells in vitro

**AT McGown[1], G Jayson[2], GR Pettit[3], MS Haran[1], TH Ward[1] and D Crowther[2]**

[1]Paterson Institute for Cancer Research and [2]Department of Medical Oncology, Christie Hospital NHS Trust, Manchester M20 9BX, UK; [3]Cancer Research Institute, Arizona State University, Tempe, Arizona, USA

**Summary** This study shows that combinations of bryostatin 1, a novel modulator of protein kinase C currently under clinical evaluation, with the anti-oestrogenic agent tamoxifen caused a large synergistic enhancement of growth inhibition in P388 cells in vitro. The growth-inhibitory effects of bryostatin 1 in the presence of non-inhibitory concentrations of tamoxifen were increased by approximately 200-fold, whereas growth inhibition by tamoxifen in the presence of non-inhibitory concentrations of bryostatin 1 were increased over 30-fold. These data have been confirmed by isobologram analysis. The precise mechanism underlying this effect is unknown, although preliminary data implicating protein kinase C is presented. The magnitude of this synergistic effect, together with evidence of clinical responses seen when these agents were given sequentially in ovarian cancer, merits further study.

**Keywords:** bryostatin; tamoxifen; synergy; protein kinase C

Bryostatin 1 is a macrocyclic lactone isolated from the marine invertebrate *Bugula neritina*, a member of the phylum Ectoprocta (Pettit et al, 1982). Bryostatin 1 exerts a wide variety of biological effects, including antineoplastic activity, immunoenhancing effects and haemopoietic stimulation. Bryostatin 1 showed significant activity in vitro against several cell lines, including leukaemia, lymphomas, melanoma, lung and renal tumours (Pettit et al, 1982; Dale and Gescher, 1989; Hornung et al, 1992). Although the precise mechanism of action of bryostatin 1 is unknown, it is believed that the agent modulates protein kinase C (PKC) (Berkow and Kraft, 1985).

The PKC proteins are a family of serine–threonine kinases that are involved in a range of cellular processes, including growth and differentiation (Azzi et al, 1992; Gescher, 1992). The precise mechanism by which activation of PKC can elicit such a variety of biological responses is unknown. However, it is likely that differences in activation, down-regulation and intracellular translocation of individual PKC isoforms after activation may give rise to this diversity of effects.

Bryostatin 1 shares many of the biological effects of known activators of PKC, such as the phorbol esters (Dale and Gescher, 1989). It has been shown that cells made resistant to bryostatin 1 show reduced expression of PKC and decreased PKC activity (Prendiville et al, 1994). A comparison of the effects of bryostatin 1 and the phorbol esters, however, shows several important differences. Bryostatin 1 does not induce differentiation in human bronchial epithelium (Jetten et al, 1989) and is not a tumour promoter. Indeed, bryostatin 1 has been shown to inhibit the tumour-promoting properties of phorbol esters (Hennings et al, 1987). These differences may arise because of differential activation, intracellular distribution or stabilization of the various isoforms that make up the PKC family.

*Received 6 March 1997*
*Revised 11 June 1997*
*Accepted 18 June 1997*

*Correspondence to:* AT McGown

Tamoxifen, a widely used anti-oestrogen, is also known to interact with PKC. However, unlike bryostatin 1, tamoxifen is an inhibitor of these kinases (O'Brian et al, 1988).

A phase I trial of bryostatin 1 (Jayson et al, 1995) showed responses in ovarian cancer. After relapse, these patients showed further responses to tamoxifen. These observations were unexpected and led to this investigation into a possible interaction between these agents. The clinical situation cannot be easily emulated, and a simplified study combining these agents in vitro was therefore undertaken. Both these agents are known to modulate PKC, and therefore the role of these kinases was also investigated.

### MATERIALS AND METHODS

The cell lines used in this study were P388 (murine lymphocytic leukaemia), P388 BR/D, a bryostatin-resistant cell line derived from the parental P388 cells (Prendiville et al, 1994), A2780 human ovarian and the human breast cell lines, MCF-7 and BT-20. These had been screened for mycoplasma and were free of infection. All chemicals and drugs were obtained from reputable sources and were of the highest quality available. Bryostatin 1 was isolated in the laboratory by one of the authors (GR Pettit). Oestrogen and progesterone receptor levels were measured by the clinical endocrinology service at the Christie Hospital, Manchester, UK, using commercial ERICA and PgRICA kits (Abbott Laboratories, UK).

Growth inhibition was determined using the MTT assay (Vistica et al, 1991) with, unless otherwise stated, continuous exposure to the drug. The analysis of the effect of drug combinations was carried out by the isobologram method (Steel and Peckham, 1979).

PMA (phorbol 12-myristate 13-acetate)-activated PKC activity was assayed by phosphorylation of an acetylated peptide based on myelin basic protein, Ac-MBP (4–14), (Yashuda et al, 1990), as described previously (Prendiville et al, 1994). P388 cells (approximately $1 \times 10^7$ per assay) were rapidly pelleted, washed with ice-cold phosphate-buffered saline, resuspended in ice-cold extraction buffer (20 mM Tris, pH 7.5, 0.5 mM EDTA, 0.5% Triton X-100 and

**Table 1** The growth-inhibitory effects of bryostatin 1, phorbol 12-myristate 13-acetate (PMA), staurosporine and tamoxifen

| Cell line | Drugs | $IC_{50}$ |
|---|---|---|
| P388 11) | Bryostatin 1 | 34.5 ± 24.1 ng ml⁻¹ (*n* = |
| P388BR/D | Bryostatin 1 | > 1 µg ml⁻¹ |
| P388 | PMA | 26 ± 13 ng ml⁻¹ (*n* = 3) |
| P3988 BR/D | PMA | > 1 µg ml⁻¹ |
| P388 | Staurosporine | 8.2 ± 4.1 ng ml⁻¹ (*n* = 3) |
| P388BR/D | Staurosporine | 12.2 ± 4.5 ng ml⁻¹ (*n* = 2) |
| P388 | Tamoxifen | 3.5 ± 1.0 µg ml⁻¹ (*n* = 8) |
| P388BR/D | Tamoxifen | 7.6 ± 5.2 µg ml⁻¹ (*n* = 3) |

**Figure 1** Isobologram analysis of the effect of combinations of bryostatin 1 and tamoxifen on the growth of P388 cells in vitro. The data points represent the $IC_{50}$ concentration (concentrations required to produce a 50% inhibition of growth). The shaded area represents the envelope of additivity as calculated by methods I and II (Steel and Peckham, 1979)

25 ug ml⁻¹ aprotinin and leupeptin) and sonicated. PKC was partly purified by binding to DEAE (diethylaminoethyl) cellulose, which had been equilibrated with ice-cold wash buffer (20 mM Tris, pH 7.5, 0.5 mM EDTA and 0.5 mM EGTA). PKC was collected as a single fraction with ice-cold elution buffer (20 mM Tris, pH 7.5, 0.5 mM EDTA, 0.5 mM EGTA, 10 mM mercaptoethanol and 0.2 M sodium chloride). Enzyme activity was assayed in triplicate after a 5-min incubation at 30°C in a reaction mixture containing 20 mM Tris, pH 7.5, 20 mM magnesium chloride, 1 mM calcium chloride, 20 µM ATP, 0.2 µCi of [γ-$^{32}$P]ATP, 50 µM Ac-MBP (4–14), 0.25 mM EDTA, 0.25 mM EGTA, 5 mM β-mercaptoethanol and 0.1 M sodium chloride with 10 µM PMA, 0.28 mg ml⁻¹ phosphatidylserine and Triton X-100 mixed micelles. Negative control levels were assayed by incubation with 20 µM PKC inhibitor [PKC (19–36)]. Reactions were terminated by spotting aliquots of the reaction mixture onto individual phosphocellulose discs, which were washed with 1% (v/v) phosphoric acid and water to remove non-incorporated [γ-$^{32}$P]ATP. Each phosphocellulose disc was placed in a scintillation vial with scintillation fluid (10 ml) for measurement of radioactivity in a Beckman (USA) LS 1801 scintillation counter. Conditions were adjusted to ensure that the reaction was linear with respect to time of incubation and concentration of cells. This assay has previously shown excellent agreement with phorbol binding assays (Prendiville et al, 1994).

### RESULTS

The effect of bryostatin 1, tamoxifen, staurosporine and PMA on the growth of tumour cells in vitro is shown in Table 1. It can be seen that the bryostatin-resistant cell line (P388BR/D) is cross-resistant to the PKC activator PMA, but not to tamoxifen.

Combinations of bryostatin 1 and tamoxifen exhibited a marked synergistic inhibition of the growth of P388 cells. Figure 1 shows that 1 µg ml⁻¹ tamoxifen potentiated the effect of bryostatin 1 more than 200-fold while 1 ng ml⁻¹ bryostatin 1 potentiated the effect of tamoxifen by more than 30-fold. This isobologram shows the effects of combinations of bryostatin 1 and tamoxifen. The data are the $IC_{50}$ concentrations of each drug both individually (bryostatin, 39 ng ml⁻¹; tamoxifen, 4 µg ml⁻¹) and in combination. If the agents were simply acting additively then the combination of drug concentrations that would cause a 50% decrease in cell growth would fall on a straight line joining the individual $IC_{50}$ values. However, non-linearity of survival curves generates an 'envelope of additivity'

**Table 2** Effect of drug combinations on the growth of cells in vitro

| Cell line | Drug 1 | Drug 2 | Enhancement of growth inhibition |
|---|---|---|---|
| P388 | Bryostatin 1 (1 ng ml⁻¹) | Tamoxifen | 34 |
| P388 | Tamoxifen (1 µg ml⁻¹) | Bryostatin | 200 |
| P388 | Staurosporine (1 ng ml⁻¹) | Tamoxifen | 1.2 |
| P388 | Staurosporine (1 ng ml⁻¹) | Bryostatin | 1.7 |
| P388 | PMA (10 ng ml⁻¹) | Tamoxifen | 25 |
| P388 | Tamoxifen (1 µg ml⁻¹) | PMA | 3.9 |
| P388BR/D | Bryostatin 1 (100 ng ml⁻¹) | Tamoxifen | 1.0 |

The final column represents the $IC_{50}$ of drug 2 (alone) divided by the $IC_{50}$ of drug 2 when given in combination with a non-inhibitory concentration of drug 1. The larger this value the greater the toxicity of the combination.

© Cancer Research Campaign 1998

Table 3 Effect of bryostatin 1 and tamoxifen, alone and in combination, on the PMA-activated PKC activity of P388 cells.

| Bryostatin 1 (ng $ml^{-1}$) | Tamoxifen (μg $ml^{-1}$) | Growth inhibition (%) | Relative PKC activity ($t$ = 24 h) |
|---|---|---|---|
| – | – | 0 | 1.0 |
| 0.1 | – | 0 | 28 ± 6 |
| 1 | – | 0 | 62 ± 8 |
| 10 | – | 30 | 0.6 ± 0.8 |
| 30 | – | 50 | 0.5 ± 0.5 |
| – | 0.2 | 0 | 52 ± 7 |
| – | 1 | 15 | 90 ± 11 |
| – | 4.1 | 50 | 1.2 ± 1 |
| 0.1 | 1 | 20 | 157 ± 27 |
| 1 | 0.2 | 25 | 8 ± 1 |
| 10 | 4 | > 90 | 0 |
| 30 | 1 | > 90 | 0 |

All PKC activities are expressed relative to control P388 cells. The results are expressed as mean ± s.d. of triplicate samples. Growth inhibition values were calculated from the isobologram curves. The activity of control cells was 0.133 ± 0.041 ($n$ = 3) pmol $^{32}P$ incorporated per μg protein.

(shaded area). If a combination of agents shows synergy (as in this case), the curve will lie below the envelope of additivity.

It was also demonstrated that the synergistic effect was maintained even when drugs were given sequentially at times up to 24 h apart (data not shown).

No synergy between tamoxifen and bryostatin 1 was observed when the P388BR/D (bryostatin-1-resistant) cells, which have reduced PKC expression, were incubated with tamoxifen in the presence of bryostatin 1 at concentrations up to 100 ng $ml^{-1}$. ($IC_{50}$ tamoxifen alone, 5.4 μg $ml^{-1}$; $IC_{50}$ tamoxifen in the presence of 100 ng $ml^{-1}$ bryostatin 1, 5.1 μg $ml^{-1}$) (Table 2).

The PKC activator phorbol 12-myristate 13-acetate (PMA) also potentiated the growth inhibitory effects of tamoxifen (Table 2). This potentiation (25-fold) was similar with bryostatin 1 (34-fold) under similar experimental conditions. However, tamoxifen at the non-inhibitory concentration of 1 μg $ml^{-1}$ caused only a modest (four-fold) increase in the growth-inhibitory effects of PMA compared with that observed with bryostatin 1 (200-fold, Table 2). Interestingly, the PKC inhibitor staurosporine did not enhance the activity of bryostatin 1 or tamoxifen (Table 2).

The roles of oestrogen and oestrogen receptors in this synergy were studied. Two cell lines with different oestrogen receptor status (MCF-7 receptor positive and BT-20 receptor negative) were shown to have differential responses to tamoxifen ($IC_{50}$ 0.7 μg $ml^{-1}$ and 3.3 μg $ml^{-1}$ respectively) but were insensitive to the growth-inhibitory effects of bryostatin 1 ($IC_{50}$ > 100 ng $ml^{-1}$). Simultaneous incubation with tamoxifen (1 μg $ml^{-1}$) did not result in growth inhibition by bryostatin 1 in either cell line at concentrations up to 100 ng $ml^{-1}$. Combinations of oestrogen and bryostatin 1 also showed no enhancement of growth inhibition in P388 cells. Furthermore, an analysis of P388 cells showed no detectable oestrogen or progesterone receptor. These data provide evidence that oestrogen and the oestrogen receptor are not involved in the synergy.

The role of PKC in the enhancement of drug sensitivity was investigated. PKC activity attributable to PMA was measured 90 min and 24 h after treatment with tamoxifen and bryostatin 1. The concentrations of drugs used were chosen to produce a range of growth-inhibitory effects when given either as single agents in combination (Table 3). All combinations and each individual drug ablated PKC activity 90 min after addition of drug. However, the PKC activity in cells treated with non- or weakly inhibitory concentrations of drugs showed elevated PKC activities 24 h after treatment (Table 3). This elevation of PKC activity was substantial and ranged from a 28-fold increase (bryostatin 1, 0.1 ng $ml^{-1}$) to a 90-fold increase for tamoxifen (1 μg $ml^{-1}$). The relatively non-inhibitory combination of drugs (bryostatin 0.1 ng $ml^{-1}$, tamoxifen 1 μg $ml^{-1}$) showed an almost 160-fold increase in PKC activity at 24 h compared with untreated (control) cells, whereas more growth-inhibitory combinations showed a loss of PKC activity at 24 h.

### DISCUSSION

Bryostatin 1 is believed to act by modulation of cell signalling via an interaction with PKC (Berkow and Kraft, 1985). PKC is an attractive target for cancer chemotherapy as these kinases play a central role in many cellular processes, including cell growth and division. PKC consists of a family of serine–threonine kinases that are activated by a number of factors. Activation is followed by translocation and down-regulation. The fate of individual isoforms is cell line dependent and influenced by a number of parameters, including the nature of the activating species.

Phase I trials of bryostatin 1 were undertaken in Manchester and Oxford, UK, under the sponsorship of the Cancer Research Campaign (Philip et al, 1993; Prendiville et al, 1993; Jayson et al, 1995). In the third of these phase I trials, bryostatin 1 was given as a 24-hour intravenous infusion, weekly for 8 weeks. The dose-limiting toxicity was myalgia, and this limited the recommended dose for phase II evaluation to 25 μg $ml^{-2}$ per week. Four responses were seen ($n$ = 19), with two (one partial and one minor) in women who had been heavily pretreated with chemotherapy for advanced ovarian carcinoma. Disease progression was noted in these patients 4 months and 6 months after completion of bryostatin 1 treatment. The patient who showed the minor response to bryostatin 1 was treated with intravenous paclitaxel, which afforded a further partial response of 4 months duration. Both patients were subsequently treated with tamoxifen (20 mg $day^{-1}$ for 6 months); the patients who had initially showed a partial response to bryostatin 1 also achieved a radiological partial response to tamoxifen of 14 months duration, while the second patient also showed stabilization of disease for 10 months.

© Cancer Research Campaign 1998

The response rate in patients with chemoresistant ovarian carcinoma treated with tamoxifen (20 mg $day^{-1}$) is 7% (Ahlgren et al, 1993). Thus, the responses, initially to bryostatin 1 and subsequently to tamoxifen, seen in these patients, although anecdotal, were unexpected and prompted this investigation into combining these agents. The clinical situation, in which bryostatin 1 and tamoxifen were given sequentially and some months apart, cannot be emulated in vitro. This initial study was carried out using combinations of these agents added simultaneously in vitro. Both agents are known to interact with PKC and the initial hypothesis was that both agents may exert their growth-inhibitory effects by interaction with a common isoform or a series of isoforms that are expressed within responsive tumours. In order to examine this, an in vitro study was designed using the bryostatin-sensitive murine P388 cell line and its bryostatin-resistant subclone (P388 BR/D), which has been shown to express reduced levels of PKC (Prendiville et al, 1994).

It was observed that addition of combination of bryostatin 1 and tamoxifen to P388 cells caused increased growth inhibition (Figure 1). Tamoxifen can reduce the concentration of bryostatin 1 required to inhibit growth in the P388 cell line by over two orders of magnitude. Similarly, bryostatin 1 can enhance the growth-inhibitory effects of tamoxifen by over 30-fold. It is unclear why there is a difference in the magnitude of the synergy (200-fold compared with 30-fold), depending upon which drug is added at the non-inhibitory concentration. Interestingly, the synergistic effect was elicited irrespective of whether the agents were added simultaneously or up to 24 h apart.

Evidence for the involvement of PMA-activated PKC in this synergy is seen in the bryostatin-resistant P388 BR/D cell line. This line has been shown to have reduced (95% reduction) PKC activity and isoenzyme expression (Prendiville et al, 1994). No synergy was observed in this cell line. Further evidence for the involvement of PKC can be seen through the use of PMA (Table 2). This potent activator of PKC enhanced the activity of tamoxifen to a level (25-fold) similar to that for bryostatin 1 (34-fold). However, tamoxifen had only a modest (fourfold) effect on the growth-inhibitory properties of PMA. This difference between PMA and bryostatin 1 is not understood but may arise by modulation of different PKC isoforms or by altered translocation and down-regulation. Bryostatin 1 and PMA have been shown to produce different effects on these processes (Levine et al, 1991; Hocevar et al, 1992; Kennedy et al, 1992).

The lack of effect of the PKC inhibitor staurosporine on the growth inhibition induced by bryostatin 1 or tamoxifen does not support a role for PKC. However, the complexity and diversity of the PKC family and the cell line-specific effects observed with PKC modulators may explain the lack of effect of this agent.

Under the conditions used in this study, tamoxifen did not induce sensitivity to bryostatin 1 in the MCF-7 and BT-20 cell lines. Thus, it is unlikely that oestrogen receptor status is a major factor in the observed synergy, as neither the MCF-7 nor the BT-20 cell lines (which are classical receptor-positive and receptor-negative cell lines, exhibiting the expected differential effects towards tamoxifen) showed increased sensitivity when treated with combinations of bryostatin 1 and tamoxifen. Further evidence against hormonal involvement is the lack of detectable oestrogen receptor expression in the P388 cell line.

The known interactions of both tamoxifen and bryostatin 1 with PKC make these signalling pathways potential mechanisms underlying this synergy. Treatment of P388 cells with bryostatin 1 and tamoxifen caused large changes in the PMA-activated PKC activity, as determined by incorporation of $^{32}P$ into the synthetic peptide (Table 3). There was a loss of PKC activity 90 min after addition of drugs. This could arise either by tamoxifen-mediated inhibition of PKC or by down-regulation of PKC by bryostatin 1. A similar rapid loss of PKC has been observed in several cell lines after treatment with bryostatin 1 (Kennedy et al, 1992). A large increase in PKC activity was observed 24 h after treatment with non-toxic levels of bryostatin 1 and tamoxifen. However the use of higher, more growth-inhibitory drug concentrations resulted in a sustained loss of PKC activity. The mechanism underlying these changes in PKC activity is currently under investigation. Work is currently ongoing to determine the fate of individual isoforms of PKC after treatment with these agents.

The potentiation of the growth-inhibitory effects of bryostatin 1 and tamoxifen is large and, if reproduced in other cell lines and more importantly in vivo, may have clinical applications. The present study shows that the P388 cell line, which is sensitive to bryostatin 1, experiences a substantial (200-fold) enhancement of growth inhibition when bryostatin 1 and tamoxifen are used in combination. Preliminary results indicate that PKC may be involved in this synergy. Extrapolation of this experimental study to the clinical observations must be approached with caution, particularly because of the differences in scheduling. Further studies to elucidate the roles of activation and translocation of individual PKC isoforms are needed to fully elucidate the involvement of these enzymes in this process.

### ACKNOWLEDGEMENT

This work was supported by the Cancer Research Campaign.

### REFERENCES

Ahlgren JD, Ellison NM, Gottlieb RJ, Laluna F, Lokich JJ, Sinclair PR, Veno W, Wampler GL, Yeung KY, Alt D and Fryer JG (1993) Hormonal palization of chemoresistant ovarian cancer: three consecutive Phase II trials of the mid-Atlantic oncology program. *J Clin Oncol* **11**: 1957–1968

Azzi A, Boscoboinik D and Henxey C (1992) The protein kinase family. *Eur J Biochem* **208**: 547–557

Berkow RL and Kraft AS (1985) Bryostatin 1, a non-phorbol macro cyclic lactone, activates intact human polymorphonuclear leukocytes and binds to the phorbol ester receptor. *Biochem Biophys Res Commun* **131**: 1109–1116

Dale IL and Gescher A (1989) Effects of activators of protein kinase C, including Bryostatins 1 and 2 on the growth of A549 human lung carcinoma cells. *Int J Cancer* **43**: 158–163

Gescher A (1992) Towards selective pharmacological modulation of protein kinase C – opportunities for the development of novel anti-neoplastic agents. *Br J Cancer* **66**: 10–19

Hennings H, Blumberg PM, Pettit GR, Herald CL, Shores R and Yuspa SH (1987) Bryostatin 1, an activator of protein kinase C, inhibits tumour promotion by phorbol esters in SENCAR mouse skin. *Carcinogenesis* **8**: 1343–1346

Hocevar BA, Morrow DM, Tykocinski ML and Fields AP (1992) Protein kinase C isotypes in human erythroleukemia cell proliferation and differentiation. *J Cell Science* **101**: 671–679

Hornung RL, Pearson JW, Beckwith M and Longo DL (1992) Preclinical evaluation bryostatin as an anticancer agent against several murine cell lines: *in vitro* versus *in vivo* activity. *Cancer Res* **52**: 101–107

Jayson GC, Crowther D, Prendiville J, McGown AT, Scheid C, Stern P, Young R, Brenchley P, Chang J, Owens S and Pettit GR (1995) A Phase I trial of Bryostatin 1 in patients with advanced malignancy using a 24 hour intravenous infusion. *Br J Cancer* **72**: 461–468

Jetten AM, George MA, Pettit GR and Rearick JI (1989) Effects of Bryostatin 1 and retenoic acid on phorbol ester and diacylglycerol induced squamous differentiation in human tracheobronchial epithelial cells. *Cancer Res* **49**: 3990–3995

© Cancer Research Campaign 1998

Kennedy MJ, Prestigiocoms LJ, Tyler G, May WS and Davidson NE (1992) Differential effects of Bryostatin 1 and phorbol ester on human breast cancer cell lines. *Cancer Res* **52**: 1278–1283

Levine B, May W, Tyler P and Hess A (1991) Response of JURKAT T cells to phorbol esters and Bryostatin 1; development of distinct functional responses and changes in protein kinase C activity. *J Immunol* **147**: 3474–3481

O'Brian CA, Housey GM and Weinstein IB (1988) Specific and direct binding of protein kinase C to an immobilized Tamoxifen analogue. *Cancer Res* **48**: 3626–3629

Pettit GR, Herald CL, Doubek DL and Herald DL (1982) Isolation and structure of Bryostatin 1. *J Am Chem Soc* **104**: 6846–6848

Philip PA, Rea D, Thavasu P, Carmichael J, Stuart NSA, Rockett H, Talbot DC, Ganesan T, Pettit GR, Balkwill F and Harris AL (1993) Phase I study of Bryostatin 1: assessment of interleukin-6 and tumour necrosis factor *in vivo*. *J Natl Cancer Inst* **85**: 1812–1818

Prendiville J, Crowther D, Thatcher N, Woll PJ, Fox BW, McGown AT, Testa, N, Stern P, McDermott R, Potter M and Pettit GR (1993) A Phase I study of intravenous Bryostatin-1 in patients with advanced cancer. *Br J Cancer* **68**: 418–424

Prendiville J, McGown AT, Gescher A, Dickson AJ, Courage C, Pettit GR, Crowther D and Fox BW (1994) Establishment of a murine leukaemia cell line resistant to the growth inhibitory effect of Bryostatin 1. *Br J Cancer* **70**: 573–578

Steel GG and Peckham MJ (1979) Exploitable mechanisms in combined radiotherapy–chemotherapy: the concept of additivity. *Int J Radiat Oncol Biol Phys* **5**: 85–91

Vistica DT, Skehan P, Scudiero D, Monks A, Pittman A and Boyd MR (1991) Tetrazolium-based assay for cellular viability. A critical examination of selected parameters affecting formazan production. *Cancer Res* **51**: 2515–2520

Yashuda I, Kishimato A, Tanaka S, Tominaga M, Sakurai A and Nishizuka Y (1990) A synthetic peptide for selective assay of protein kinase C. *Biochem Biophys Res Commun* **166**: 1220–1225

© Cancer Research Campaign 1998